# Polycentrism in Global Health Governance Scholarship: Comment on "Four Challenges That Global Health Networks Face"

**DOI:** 10.15171/ijhpm.2017.64

**Published:** 2017-05-23

**Authors:** Jale Tosun

**Affiliations:** Institute of Political Science, Heidelberg University, Heidelberg, Germany.

**Keywords:** Coalition-Building Tactics, Global Health Networks, Governance, Polycentrism

## Abstract

Drawing on an in-depth analysis of eight global health networks, a recent essay in this journal argued that global health networks face four challenges to their effectiveness: problem definition, positioning, coalition-building, and governance. While sharing the argument of the essay concerned, in this commentary, we argue that these analytical concepts can be used to explicate a concept that has implicitly been used in global health governance scholarship for quite a few years. While already prominent in the discussion of climate change governance, for instance, global health governance scholarship could make progress by looking at global health governance as being polycentric. Concisely, polycentric forms of governance mix scales, mechanisms, and actors. Drawing on the essay, we propose a polycentric approach to the study of global health governance that incorporates coalitionbuilding tactics, internal governance and global political priority as explanatory factors.

Starting as a system with the World Health Organization (WHO) together with a handful of influential states as the key actors, the global health regime has transformed in such a fashion that it now comprises a whole range of (global) networks and (global) public-private partnerships.^1-3^ The rising complexity of the global health regime raises a number of questions that warrant attention from both public health scholars and practitioners. What is the role of global health networks in global health governance? Under what conditions are global health networks effective? What is the legitimacy of global health networks? These three research questions are exactly those put forth in Jeremy Shiffman’s^4^ recent essay on the four challenges global health networks face.

Shiffman – who considers global networks to be “webs of individuals and organizations with a shared concern for a particular condition” (p. 183) – presents eight global health networks and discusses to what extent they were effective in bringing about collective action. The author identifies the generation of attention and allocation of resources to be critical for determining the networks’ effectiveness. Attention and resources are affected by four challenges that global health networks are faced with. The first challenge refers to the process of generating consensus on what the problem is and how it should be addressed (*problem definition*). The second challenge refers to the (strategic) portrayal of an issue with the aim to induce external audiences to act (*positioning*). The third challenge refers to the process of forging alliances with these external audiences (*coalition-building*). The fourth challenge consists of establishing institutions that facilitate collective action (*governance*). Building on Shiffman’s insightful essay, this commentary strives to advance the argument that global health governance scholarship could make progress by more explicitly looking at global health governance as polycentric.

Research on other policy problems such as climate change^5-9^ has adopted the perspective of polycentric governance, which especially in its current form is closely associated with the work of Elinor Ostrom.^10^ Briefly, polycentric governance posits that solutions to global challenges must be formulated by multiple, formally independent decision-making authorities operating at multiple scales. Sovacool^11^ conceives polycentrism to not only include multiple scales (eg, the international level), but also mechanisms (eg, centralized command and control regulations), and actors (eg, government institutions).

When looking at the 2030 Agenda for Sustainable Development, the notion of polycentric governance seems to reflect the empirical reality. Among the Sustainable Development Goals adopted in 2015, Goal 3 is about ensuring health and well-being for all at every stage of life.^12,13^ While this goal is aligned with a number of more specific targets and indicators, there is no uniform procedure for how ‘health for all’ is to be reached.^14^ A probable scenario is that public actors at the international, regional, national, and subnational level together with private actors will design and implement policy actions for attaining Goal 3. The simultaneous existence of a range of policy actions by multiple actors at different scales corresponds to the definition of polycentric governance.

In perceiving health governance to be polycentric, different and arguably more innovative research questions could emerge. For example, instead of concentrating on failure and fragmentation, this new perspective invites questions about the emergence and effectiveness of global health networks – a research perspective in fact already adopted by Shiffman and colleagues.^15^

Polycentric governance is likely to produce effective, equitable, and sustainable outcomes as this governance form increases mutual trust between the individual actors as well as stimulates communication processes and cooperation, which can then lead to policy learning.^8,10^ For the same reasons, polycentric governance can possess some weaknesses, as it increases transaction costs, creates organizational redundancies, and can lead to tension and conflict among actors.^9^ Moreover, polycentric governance can facilitate competition between different actors and initiatives and induce strategic action.^6^

This latter point makes a strong case for the need to understand how global policy networks influence the definition of problems, how they position themselves on these problems, how they build coalitions with external actors, and which institutions they choose to govern their governance activities. When examining global health networks from this position, the four ‘challenges’ correctly identified by Shiffman^4^ become analytical dimensions that allow for a comprehensive and comparative assessment of what the individual global health networks look like and how these features are likely to affect their role in a polycentric system.

Shiffman’s essay,^4^ however, does not explore the relationship between the individual global policy networks in order to explain their effectiveness. Yet it is conceivable that the effectiveness of global health networks also depends on the extent to which they compete with one another on the same scale (eg, the global level) or on different scales (eg, the national level). Competition among health networks crucially depends on how the problem addressed by the respective networks is defined. Problem definition is a consequential endeavor since it defines the ‘scope of conflict.’^16,17^ The individual networks must decide on whether they want to expand the scope of the conflict or contain it. When defining a problem in the broadest possible way, this will increase attention, but it is also likely to increase the definitional overlap with other networks, which can then lead to competition among networks. At the same time, it can also generate (greater) conflict within the network itself as different groups argue for their preferred problem definitions and seek to make them a priority issue. When defining a problem more narrowly, competition among networks is likely to be reduced, but this will also reduce the conflict’s level of attention and the possibility to mobilize support for the network’s goals from inside and outside the network.

While the treatise of competition between health networks is a proposal for expanding Shiffman’s contribution,^4^ there is also potential for streamlining the argument. More precisely, the dimensions of positioning and coalition-building seem suitable to be merged with problem definition. This modification seems reasonable since problem definition and positioning are two very closely interlinked processes. Assuming that global health networks are rational collective actors, they will attempt to define a problem in such a fashion that it corresponds to their positioning on it. Likewise, problem definition is key for forging an alliance with external actors as it is through the definition of the scope of the conflict that some actors are brought into the process and others are excluded from it. From this, it follows that these three concepts – problem definition, positioning, and coalition-building – are interdependent processes and that therefore, the outcomes of one dimension will affect the outcomes of the other. For example, if a problem is defined too narrowly, it will also be portrayed as a rather narrow issue, which will make it rather difficult to forge alliances with a great number of external actors.

Consequently, Shiffman’s framework could just go with ‘coalition-building tactics’ along with the dimension referring to the governance inside the global health networks. Another factor that is mentioned in the table on page 185, but is not flagged as a constitutive element of the framework, is the global policy priority given to the problems the individual global health networks deal with. This factor is worth discussing more systematically since coalition-building tactics and governance are endogenous to networks, whereas global policy priority is exogenous to them, which allows for proposing a more complete explanatory model. Global policy priority as a concept also aligns well with polycentric governance as it offers an opportunity structure for policy actions.

In closing, Shiffman^4^ discusses the legitimacy of global health networks, which has also been addressed by studies adopting the perspective of polycentric governance.^9^ Shiffman’s treatise on this aspect is particularly illuminating since he does not engage in a normative debate, but posits two questions that can be evaluated empirically. The first one is to what extent the deficiencies of international organizations and national governments in addressing pressing health problems justify the existence of global health networks (p. 188). This question again reflects the global governance perspective underlying this article as it stresses the failure of a centralized policy response at the international and national level. However, this perspective is well taken as it does not draw on democratic legitimacy, but taps into the conditions under which the authority of networks is accepted.^5^ The second question Shiffman posits is to what extent global health networks actually exert power without possessing legitimate authority, which offers a promising starting point for future research that may approach health governance from the perspective of polycentric governance.

Overall, Shiffman’s essay includes a number of analytical concepts that allow for more explicitly looking at global health governance as polycentric. The fact that Shiffman analyzes global health networks and that these constitute empirical realities already indicates that global health governance is polycentric. Moreover, global health governance is characterized by processes that take place at the same time, but perhaps at different governance scales, which again aligns well with the concept of polycentrism. However, the relevant literature does not use this term and does not refer to the literature on polycentrism that deals with other topics in global governance such as climate change. Yet it seems that global health governance scholarship can benefit from embracing this concept, which should be easy to attain since the literature already operates with numerous concepts that are related to polycentrism.^1-3^ One of the key advantages would be the posing of new research questions. Another advantage can be seen in the potential to integrate the literature on global health governance with strands of scholarship on other topics that, however, are also of a transnational nature and face the same or at least similar opportunities and constraints for governance action.

## Acknowledgements

This research is the outcome of a research fellowship at the Marsilius Centre for Advanced Study at Heidelberg University, Heidelberg, Germany, during which the author collaborated with Albrecht Jahn, Bernd Grzeszick, and Gorik Ooms. Jeremy Shiffman was kind enough to comment on an earlier version of this commentary. Rebecca Abu Sharkh and Rana Chen deserve credit for language editing. Laura Zöckler provided valuable research assistance.

## Ethical issues

Not applicable.

## Competing interests

Author declares that she has no competing interests.

## Author’s contribution

JT is the single author of the paper.
